# Frequency, Characteristics, and Predictive Factors of Adverse Drug Events in an Adult Emergency Department according to Age: A Cross-Sectional Study

**DOI:** 10.3390/jcm11195731

**Published:** 2022-09-27

**Authors:** Laura Lohan, Grégory Marin, Marie Faucanie, Marion Laureau, Damien Perier, Véronique Pinzani, Isabelle Giraud, Maxime Villiet, Mustapha Sebbane, Ariane Sultan, Cyril Breuker

**Affiliations:** 1 Clinical Pharmacy Department, CHU Montpellier, University of Montpellier, 34295 Montpellier, France; 2 PhyMedExp, University of Montpellier, CNRS, INSERM, 34000 Montpellier, France; 3 Clinical Research and Epidemiology Unit, CHU Montpellier, University of Montpellier, 34295 Montpellier, France; 4 Emergency Medicine Department, CHU Montpellier, University of Montpellier, 34295 Montpellier, France; 5 Medical Pharmacology and Toxicology Department, CHU Montpellier, University of Montpellier, 34295 Montpellier, France; 6 Economic Evaluation Unit, CHU Montpellier, University of Montpellier, 34295 Montpellier, France; 7 Endocrinology-Diabetology-Nutrition Department, CHU Montpellier, University of Montpellier, 34295 Montpellier, France

**Keywords:** adverse drug event, epidemiology and detection, age factors, emergency department, patient safety, healthcare quality improvement, pharmaceutical team

## Abstract

Adverse drug events (ADEs) are a major public health concern, given their consequences in terms of morbi-mortality and associated healthcare costs. Many studies have focused on the elderly, who are considered particularly vulnerable in this respect. We aimed to determine and compare the frequency, characteristics, and predictive factors of ADEs according to age in an adult population. A prospective seven-year cross-sectional study was conducted in a university hospital emergency department. Structured medication reviews and ADE detection were performed. Patient data and ADE characteristics were collected. Descriptive statistics and logistic regression were performed in two age groups: Group 1 (age < 65 years) and 2 (age ≥ 65 years). Among the 13,653 patients included, 18.4% in Group 1 and 22.6% in Group 2 experienced an ADE. Differences were identified in terms of the ADE type (more ADEs due to noncompliance in Group 1) and ADE symptoms (greater bleeding in Group 2). In the multivariable analysis, several specific predictive factors were identified, including kidney failure and antidiabetic drug use in Group 1 and inappropriate prescription and antithrombotic treatment in Group 2. Analysis by age provided a more refined vision of ADEs as we identified distinct profiles of iatrogenesis. These results will lead to a better detection of ADEs.

## 1. Introduction

Adverse drug events (ADEs) are a major public health issue in both ambulatory and hospital settings. One of their consequences is hospital admission [1,2], particularly via the emergency department (ED) [3,4], where they may outweigh other common reasons for ED visits, like syncope or pneumonia [5]. Studies have reported that patients with ADEs in an ED have higher priority scores and worse outcomes [6], are admitted to the hospital more frequently [5,7,8] and for longer periods [7,9], and have a higher cost of care [9] than patients without ADEs. The latter two findings may also apply to inpatients experiencing medication-related problems, often with an increased risk of death [10]. These consequences impact many people, given the non-negligible frequency of medication-related problems. A recent meta-analysis estimated an 8.3% pooled prevalence of adverse drug reactions (ADRs) in primary care settings, ranging as high as 20.4% when only studies with a low risk of bias were considered [11]. Similarly, ADE frequency in inpatients up to 30% has been reported [12]. 

Given this medication-related healthcare burden—a substantial part of which is preventable [6,7,13]—many studies have been conducted to better characterize ADEs and identify the determining factors [2,4,14,15]. Notably, large-scale national surveillance initiatives have been implemented, such as NEISS-CADES [16,17,18] in the United States launched in 2003 and the MEREAFaPS project [19,20] in Italy launched in 2006. Predictive risk models and tools for use in clinical practice have also been developed [12,21]. Nevertheless, ADEs remain an ongoing problem, reflected by the title of the last World Health Organization (WHO) Global Patient Safety Challenge: “Medication Without Harm” [22]. In that year, 2017, the WHO estimated the cost of medication errors at $42 billion USD annually.

Further research into drug-related issues is therefore needed. Studies have regularly identified advanced age as a potential risk factor for ADEs [2,4,14,15], with patients 65 years and older twice as likely to be admitted to an ED unit for an ADE compared to younger patients, and seven times more likely to require hospitalization as a consequence [17]. The factors usually mentioned to explain this increased age-related risk include multimorbidity, polypharmacy, physiological change—particularly changes in drug metabolism—and cognitive impairment [23]. This overall frailty and higher vulnerability to ADEs is a public health concern, especially given the growing size of the elderly population. Several studies have thus focused on this population, developing lists of inappropriate medications [24] and investigating the prevalence, main features, and risk factors for drug-related problems in elderly patients [25,26]. However, to our knowledge, none has included comparisons with a younger population. Yet, a comparison of ADE data between age groups would likely broaden our understanding of the age-related specificities of ADEs and thereby enable more effective adaptations of the means for detection. 

Our objectives were to determine and compare the frequency, characteristics, and predictive factors of ADEs according to age in an adult population. We used data from our 7-year ADE observatory to explore this issue. 

## 2. Materials and Methods

### 2.1. Study Design

This study was part of the prospective ADEsED study that was conducted from November 2011 to November 2018 in the general ED of our French University Hospital (2600-bed tertiary care center). It was performed according to the World Medical Association Declaration of Helsinki, approved by the Montpellier University Hospital Institutional Review Board), and registered on ClinicalTrials.gov (NCT03442010, accessed on 25 August 2022). 

### 2.2. Study Population

Participation in this study was proposed for all adult patients (>18 years) seen by a member of the dedicated ED pharmacy team for a medication history interview. All patients who gave consent were prospectively included and followed until discharge. Non-inclusion criteria were refusal to participate, voluntary medication poisoning, and acute psychological disorders such as psychotic breaks. We defined two study groups according to age: Group 1 with patients <65 years old and Group 2 with patients ≥65 years old.

### 2.3. Intervention and Measurements

Details of the intervention have been published elsewhere [27,28]. Briefly, a member of the pharmaceutical team, which is part of the general ED staff, conducted an interview with each patient to obtain the medication history and then begin the ADE detection process. All team members had received training in the medication history-taking [29] and ADE detection and documentation [30,31]. 

The medication history process followed the WHO High 5s standard operating procedures [29]. It covered prescription drugs, self-medication, and as-needed treatments. We also collected self-reported adherence through direct questions about the possibility of omission, dosage, or duration modifications, but also open-ended discussion about prescription respect in general. The history also included information from various sources: the patient and/or relatives, patient prescription(s), the medical record, and the healthcare professionals involved in the patient’s care (including the community pharmacy). Once this information was collected, the ADE detection process began. 

For this study, ADEs were defined as unfavorable occurrences related to the use or misuse of medications [32,33]. This definition includes signs, symptoms, and laboratory abnormalities resulting from adverse drug reactions (ADRs) or noncompliance with medication prescriptions. The method for identifying and validating an ADE has been described elsewhere [27,28] and is summarized in Supplementary Figure S1. A pharmacist and an ED physician independently identified the ADEs and consulted an expert committee in cases of doubt about the diagnosis or category. The events could be qualified as “direct ADRs” (the medication was judged to be the only cause of the presented symptom) or “participating ADRs” (the medication was judged to have facilitated or aggravated the symptom) [34]. ADEs resulting from under- or overdosing of the prescribed treatment were considered inappropriate use, even when unintentional, and grouped into the category of “noncompliance with drug prescription”. ADE severity was assessed according to the Common Terminology Criteria for Adverse Events [35]. The primary outcome was the confirmed occurrence of an ADE, whether it was the reason for the ED visit.

The ADE characteristics and therapeutic data (medication name and Anatomical Therapeutic Chemical (ATC) code [36], potentially inappropriate medications (PIMs) according to published lists) obtained through the medication history and ADE detection process were prospectively recorded in an anonymized database. The PIM lists initially selected were Laroche’s list [37], the Beers Criteria [38], and the short version of the PIM-EU7 list [39]. Medications were listed as always inappropriate when the corresponding list identified them as such, or conditionally inappropriate when they could be inappropriate depending on the conditions of the drug use (such as indication, dose, adaptation to renal function, etc.). The proportion of PIMs according to these lists was counted in both age groups for comparison purposes, our objective being to verify the age-related specificity of ADE determinants. The anticholinergic potential of the recorded drugs was also assessed using the Anticholinergic Cognitive Burden Scale (ACB) [40], the Anticholinergic Drug Scale (ADS) [41], and the Anticholinergic Risk Scale (ARS) [42].

The pharmaceutical team concurrently collected sociodemographic characteristics, admission data (FRENCH triage scale level [43], reason for the ED visit), discharge disposition and clinical-biological information from the medical record and/or structured patient interview. Some comorbidities were inferred from the patient’s treatment and are referred in the results as “treated comorbidities”. For example, patients were considered to have treated diabetes when antidiabetic drugs were present in their treatment.

### 2.4. Analysis

Only patients with at least one medication were included in the analyses, and the analyses were performed separately for the two age groups. The patient characteristics are expressed as frequency and proportion for categorical variables and as means ± standard deviations (SD) for continuous variables. Within the two groups, the characteristics of patients with and without ADE occurrence were compared with Student’s *t* or the Mann-Whitney *U* test for continuous variables and with the Chi-square or Fisher exact test for categorical variables. Patient characteristics were also compared between the two groups using the same tests. 

We performed multivariable logistic regression analyses in both age groups to identify the factors possibly associated with an ADE occurrence. The variables considered for the multivariable model were those with *p*-values >0.10 in the univariate models. Parameters with a high number of missing values (>40%) were not considered. The final list of variables retained for the multivariable analysis is presented in Supplementary Table S1. After a stepwise selection of variables to statistically eliminate redundant and/or collinear variables, we kept in the final model only those variables that were significantly associated (*p*-values < 0.05) with ADE occurrence in the multivariable model. Missing data were not replaced and the absence of collinearity between the variables of interest was verified. 

Statistical analyses were performed with SAS 9.1 (SAS Institute, France), and the statistical bilateral significance threshold was set at 5%.

## 3. Results

### 3.1. Frequency of ADEs

**Figure 1:**
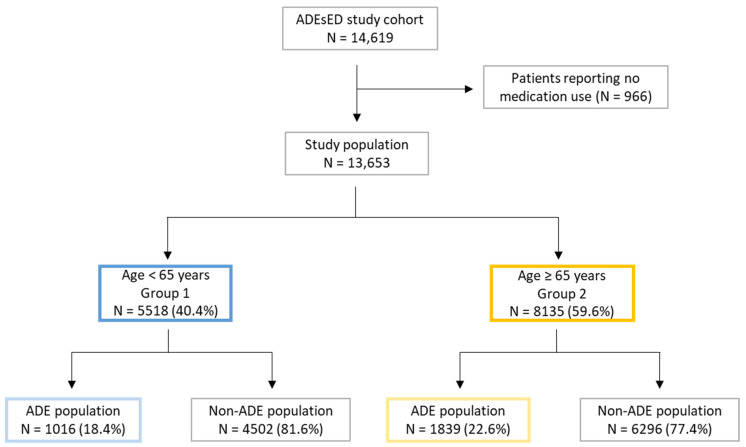
Flowchart of the study population. ADE, adverse drug event; ADEsED: adverse drug events at emergency department (ADEsED); ED, emergency department. ADEsED study is registered on ClinicalTrials.gov (NCT03442010, accessed on 25 August 2022).

The flowchart of the study is presented in Figure 1. Our study population consisted of 13,653 patients, 5518 (40.4%) in Group 1 (patients < 65 years) and 8135 (59.6%) in Group 2 (patients ≥ 65 years). Their characteristics are presented in Table 1. ADEs were detected in 20.9% of the total population, 18.4% in Group 1, and 22.6% in Group 2. These proportions significantly differed between the two age groups (*p* < 0.01).

### 3.2. Comparison of General, Clinical and Therapeutic Characteristics of ADE Patients

Many characteristics significantly differed between Groups 1 and 2 of the ADE population, as expected. Concerning general and clinical data (Table 1), the Group 2 ADE patients had higher priority scores (75.8% with levels 1 to 3 vs. 61.2%, *p* < 0.01) and were more often hospitalized (55.6% vs. 32.8%, *p* < 0.01). The main reason for the ED visits was hepatic/gastrointestinal symptoms in Group 1, whereas it was malaise and fatigue in Group 2. Except for active cancer, the Group 2 ADE patients presented more treated comorbidities and generally more clinical-biological abnormalities. 

Therapeutic data are presented in Table 2. Treatment management was very different between the two ADE age groups, with notably higher compliance in Group 2. Interestingly, this result was found regardless of the presence or absence of an ADE, with 66.0% compliance in Group 2 and 51.1% in Group 1 (*p* < 0.01). Nevertheless, the number of treatments was lower in Group 1, but with more self-medication. Only three drug classes were significantly more prescribed in the Group 1 ADE patients, namely antineoplastic agents, immunosuppressants, and anti-inflammatory products. Regardless of the list considered, PIMs were higher in Group 2 ADE patients. Notably, more than 80% of these patients had an always or conditional PIM in their treatment according to the Beers Criteria or PIM-EU7 list, with a mean number of PIMs of 1.2 ± 0.5 and 1.5 ± 0.7, respectively. The same trend was observed for anticholinergic drugs, except when the ARS score was used.

For more information on general and therapeutic data in the total population of each group, a specific table is available in the supplementary material (Supplementary Table S2).

### 3.3. Characteristics of ADEs including Medication Involved

Table 3 shows the main ADE characteristics. ADE symptoms differed greatly between the two groups. In Group 1, the main ADE symptoms were neurologic (20.0%) and gastrointestinal (17.1%), whereas bleeding was the main symptom (35.2%) in Group 2. An ADE was the reason for the ED visit in 86.1% and 72.8% of the cases, respectively. Except for direct ADRs, which were overall the most frequent type of ADE (44.9%), we observed significant differences in ADE categories, with more ADRs in Group 2 and more noncompliance in Group 1. Last, 50.4% of the Group 2 patients were hospitalized due to their ADE vs. 30.3% in Group 1 (*p* < 0.01).

**Figure 2:**
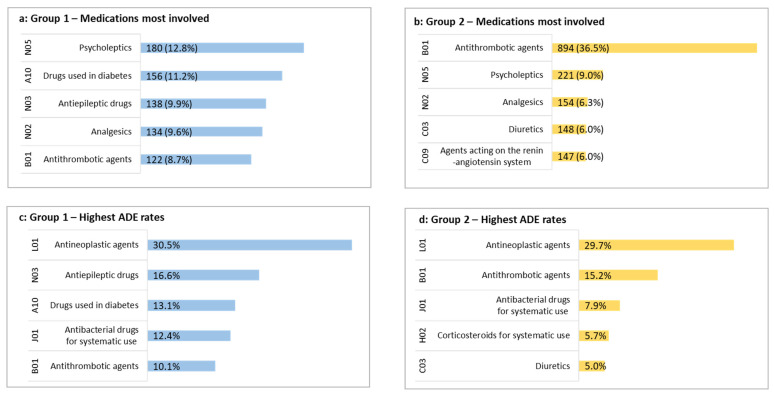
Lists of the top 5 medications most involved in ADEs and with the highest ADE rates. For medications most involved (**a**,**b**) data are presented as *n* (% of all medications involved in an ADE) for patients being less than 65 years (**a**) and more than 65 years (**b**), categorized by ATC class level 2. Medications ADE rates were determined by dividing the number of times a medication was involved in an ADE by the total number of prescriptions for that medication among included patients. ADE rates are presented as a percentage, for patients being less than 65 years (**c**) and more than 65 years (**d**), categorized by ATC class 2.

Figure 2 provides an overview of the medications most involved in ADEs. Complete data are available in Supplementary Table S3. The analysis of the ADE rate (i.e., the number of times a given medication was involved in an ADE divided by the number of times it was prescribed among included patients) is presented in Figure 2 and Supplementary Table S3.

Of the 25,130 drugs identified in the Group 1 patients’ treatment (see Supplementary Table S4), 1398 were involved in an ADE. Nervous system drugs were the first medication class involved (38.8%), including psycholeptics, antiepileptics, and analgesics. Alimentary tract and metabolism drugs were next (14.1%) and were mostly those used in diabetes (11.2%). The medications with the highest ADE rate were antineoplastic agents (30.5%) followed by antiepileptics (16.6%) and antidiabetics (13.1%).

Concerning Group 2, 2449 medications out of 63,965 were involved in an ADE. Blood and blood-forming organ drugs, particularly antithrombotic agents, ranked first. They came second in terms of the rate of medication involvement (15.2%), behind antineoplastics (29.7%).

Drugs considered to be always inappropriate ranged from 5.4% (Beers Criteria) to 11.9% (PIM-EU7 list) of all ADE-causing drugs in Group 1, and 3.5% (Laroche’s list) to 10.5% (PIM-EU7 list) in Group 2. The proportion of ADEs induced by a PIM was not significantly different between the groups, except when Laroche’s list was used (5.7% in Group 1 vs. 3.5% in Group 2, *p* = 0.02). Anticholinergic agents accounted for 4.7% (ARS) to 26.0% (ADS) of the ADEs in Group 1, and 3.7% (ARS) to 28.2% (ADS) in Group 2. Only the ACB score was discriminant between Groups 1 and 2, showing ADEs in 18.7% and 24.2% of the cases, respectively (*p* < 0.01).

### 3.4. Predictive Factors of ADEs

Table 1 and Table 2 present the results of the univariate analysis of the potential relationships between patient characteristics and ADE occurrence in both age groups. The results of the multivariable analysis are presented in Table 4. Eight variables were common to both groups. Among them, the most significant for Group 2 was bleeding as the reason for the ED visit (OR = 6.4; CI95% [5.1–8.0]). For Group 1, it was the presence of an antineoplastic agent in a patient’s treatment (OR = 4.3; CI95% [2.9–6.6]). Two independent predictors were retrieved only in patients of <65 years: kidney failure and treatment with drugs used in diabetes. Of the 10 variables observed only in patients of ≥65 years, four were the primary reason for the ED visit, negatively associated with ADE presence. The remaining six were factors associated with a greater risk of ADE. These factors were a higher priority score, two electrolyte disorders, treatment with antithrombotic agents or beta-blocking agents, and a PIM, according to at least one list.

## 4. Discussion

We compared for the first time the frequency, characteristics, and determinants of ADEs in two age groups, individuals of <65 years and ≥65 years, using data prospectively collected from over 13,000 people visiting an ED. Although ADE frequency was higher in the ≥65 population, we highlighted that in adult patients below that age, this frequency was not negligible. We showed that the ADE characteristics, in terms of symptoms, category, severity, and drugs involved, were quite different between the two age groups, suggesting distinct iatrogenic profiles. This observation was reinforced by the identification of predictive factors for ADEs specific to each group, including antidiabetic treatment in Group 1 and inappropriate prescription or antithrombotic treatment for the second.

We found an ADE frequency in the ED setting of 18.4% for patients of <65 years and 22.6% for those of ≥65 years. We selected studies to which we might best compare our study in terms of population, methodology, and type of ADE examined and found that the ADE frequencies ranged from 8.2 to 22.5% in the adult population [6,9,44,45,46,47], consistent with our results. For the frequency by age group, the figures available for the ≥65 group ranged from 14.4 to 20.0% [48,49,50]. In contrast, we were unable to find comparable data for the group between 18 and 64 years. ADEs were less frequent in this age group than in patients of ≥65. Nevertheless, the difference was not as obvious as we had expected, and frequency remained in the high range of the previously cited estimates for a population not sorted by age.

Further, we identified two distinct ADE profiles according to age. In the ≥65 group, bleeding was the predominant ADE symptom, matching the class of drugs found to be the primary cause of an ADE in this population: antithrombotics. This drug/symptom combination has been noted by studies focused on the elderly [51], the elderly subgroup of a wider population [20,52], and a population with no age specificities [46]. However, in this last study, the mean age of the population was 61.5 years, which may have led to results more reflective of the older portion of the population. Regarding the ADE category, ADRs were predominant. The medication involved had either been the sole cause of the ADR or had contributed to its occurrence as part of a multifactorial pathological condition. This observation is consistent with the overall age-related frailty already mentioned. Moreover, hospitalizations for ADEs were significantly higher than in the <65 population, which supports the finding of greater frailty, although they may also have been related to greater ADE severity. In this respect, age has been identified as a predictive risk factor for severe ADEs in some studies [8,27], but others have found no difference in the incidence of serious ADEs or drug-related hospitalizations between patients younger and older than 65 years [53].

In patients of <65 years, neurologic and gastrointestinal reactions were the two main symptoms. Although no direct comparison with the literature can be made, this is consistent with a study in which the mean age was 49.3 years [7]. Contrary to our older profile, several drug classes were concerned with no clear predominance of one over the others. First involved were the psycholeptics (12.8%), followed by antidiabetics (11.2%) and antiepileptics (9.9%). While it is unsurprising to see psycholeptics on this list [11], antiepileptics do not frequently stand out in this context, and antidiabetics are usually more likely to be associated with an ADE risk in the elderly [20,51]. These unusual results may reflect the specificity of the two age groups but may also be interpreted via the notion of noncompliance. Indeed, we included noncompliance as a category in our ADE definition, as recommended but not systematically done [7,20,49], and observed a substantial proportion of this ADE type, three times more than in the ≥65 patients. Lower compliance in general, apart from the notion of ADEs, was also noted for this group, despite fewer medications. All these particularities are very informative about iatrogenesis in the <65 population and highlight the importance of improving adherence as part of a better ADE prevention strategy.

Some ADE characteristics were common to our two groups, including the frequent involvement of psycholeptics and analgesics, in accordance with the available literature. However, is this finding a reflection of the inherent excess risk of these classes, or is it a consequence of their widespread prescription? Indeed, more than half of our participants reported using analgesics, and it is therefore unsurprising that these drugs were among the most frequently involved in ADEs. We therefore considered another parameter that we called the ADE rate, which is the ratio of the number of ADEs caused by a drug class to the number of prescriptions for that class among the included patients. This is by no means a representative rate of drug safety in the general population, but a different expression of the involvement of a drug in an ADE among the population attending our ED. We then observed that antineoplastic drugs dominated as those most likely to cause ADEs in both patient groups, even though they were behind in overall frequency, being rarely prescribed. This further explains why they have emerged poorly in large-scale studies that only list drugs that have caused an ADE [20,52]. In contrast, analgesics disappeared from the list of high-risk drugs because calculating the ADE rate resulted in a lower weight of the prescription frequency.

Our last objective was to identify the variables associated with the presence or absence of an ADE through multivariable analysis. Several of the identified independent predictive factors differed between patients of <65 years and ≥65 years. Moreover, for the factors common to both groups, the strength of the association with ADE occurrence was also often different. Thus, although common to both groups, compliance parameters stood out in patients of <65 years. Antidiabetic drugs in patient treatment were also identified as a predictive factor for ADE only in this group, reinforcing the warning not to overlook ADEs in this age group. Another predictive factor was a GFR < 60 mL/min/1.73 m^2^. This finding is interesting as the renal function might be expected to be more problematic in older patients, and it should thus alert us not to overlook GFR in younger patients. Concerning our ≥65 group, bleeding as the reason for ED visits showed the highest odds ratio. This is unsurprising given that bleeding was also the primary symptom of ADEs in this population. Moreover, blood and blood-forming organ drugs were the major medications involved in ADEs. This underlines the close links between medications, ADEs and the reasons for emergency admission. For the medications, the multivariable analysis highlighted both antithrombotics and inappropriate medications in patient treatment as specific predictive factors for ADEs. Interestingly, while the selected PIM lists showed significance in the univariate analysis of both age groups, none stood out alone in the multivariable analysis. Only the composite criteria “always inappropriate according to at least one list” was identified as an ADE predictive factor in ≥65 patients. The modest ADE prognostic power of some of the lists and the lack of the clear superiority of one list have been discussed in the literature [54]. Nevertheless, PIM prescriptions were identified as a predictive factor for ADEs, suggesting that PIMs should not be ignored in this group, especially since many of the drugs are still highly prescribed. In addition, a complementary approach to using these lists seems better than relying on only one of them.

Some limitations to our work must be emphasized. First, although we had a large number of patients, our study was monocentric. Moreover, our recruitment procedure, which was not strictly systematized, may have resulted in the inclusion of a preponderance of elderly patients. This raises questions about how representative our overall population was and how generalizable our results are. However, the analysis by age group and a large number of patients per group limited this potential overrepresentation. Moreover, another explanation for the mean age is that we included only patients reporting as-needed or chronic use of at least one medication. In addition, we chose an age cutoff of 65 years for our two groups as it is frequently used in studies on ADEs in the “elderly”, as well as for most PIM lists. It would have been interesting, however, to perform a subanalysis with several age categories in the ≥65 population to obtain more specific data, especially for the oldest patients. Finally, we did not evaluate the preventability of ADEs, which would have added value to our work. Nevertheless, some of the features of our study allowed us to glimpse this notion, such as the inclusion of noncompliance in the ADE definition and the identification of ADEs induced by PIMs.

Our study also has strengths, primarily its prospective design, the number of patients included, and the number of observations of the outcome of interest. Our rigorous approach to obtaining medication histories and detecting ADEs should also be noted. Identifying ADEs requires a proper medication history, but this information is not necessarily available or accurately reported in the ED [55,56]. Our standardized methodology guarantees that this history is (i) reliable, by combining several sources of information; (ii) exhaustive, by considering all drugs, even self-medication and over-the-counter drugs; and (iii) up to date. Regarding ADE detection, our collegial, multidisciplinary, and prospective process helped us address the known issues of under-detection and under-reporting [57,58]. The rather broad definition of ADEs is another strength of our study, as we were able to objectify important findings for the prevention of certain ADEs, particularly concerning noncompliance with treatment. Finally, we collected each patient’s medication treatment (89,095 drugs), not just the drugs that induced an ADE. This enabled us to deepen our analysis and individualize the specific ADE rate of each drug class in our population, which is unique and very informative.

## 5. Conclusions

We have shown that, although ADEs are more frequent in people of ≥65 years, ADEs in people of <65 years should not be overlooked, given their non-negligible frequency in this age group. We also identified valuable characteristics and predictive factors of ADEs in both age groups for the first time. The analysis by age provided a more refined vision as we were able to identify distinct profiles of iatrogenesis. Further work remains to be done, but these factors could be used as tools to tag patients with potentially undiagnosed ADEs and thus to set up ADE secondary prevention interventions after discharge from the ED.

## Figures and Tables

**Table 1:** Characteristics of the population by age group and presence/absence of an ADE.

	Study Population (*n* = 13,653)	Group 1			Group 2			Comparison of ADE Populations (Group 1 vs. Group 2) *p*-Value
		ADE Population (*n* = 1016)	Non-ADE Population (*n* = 4502)	*p*-Value	ADE Population (*n* = 1839)	Non-ADE Population (*n* = 6296)	*p*-Value	
Sociodemographic data								
Age (*n* = 13,653)	66.1 (±14.1)	43.8 (±14.1)	43.8 (±14.1)	0.93	81.9 (±16.9)	81.0 (14.2)	<0.01	
Gender (*n* = 13,653)—Female	7135(52.26)	488 (48.0)	2301 (51.1)	0.08	943 (51.3)	3403 (54.1)	0.04	0.1
Lifestyle (*n* = 13,620)				<0.01			0.13	<0.01
At home	12,120 (89.0)	940 (93.4)	4383 (97.7)		1515 (82.5)	5282 (83.9)		
In institution	1500 (11.0)	66 (6.6)	102 (2.3)		322 (17.5)	1010 (16.1)		
Admission data								
ED unit of inclusion (*n* = 13,635)				<0.01			<0.01	<0.01
Emergency critical care unit	455 (3.3)	42 (4.2)	76 (1.7)		120 (6.5)	217 (3.4)		
Observation emergency unit	11,983 (87.9)	861 (85.1)	4233 (94.2)		1409 (76.7)	5480 (87.1)		
Short-stay hospitalization unit	1197 (8.8)	109 (10.8)	184 (4.1)		309 (16.8)	595 (9.5)		
FRENCH Triage Scale (*n* = 13,528)				<0.01			<0.01	<0.01
Level 1	372 (2.7)	32 (3.2)	59 (1.3)		108 (5.9)	173 (2.8)		
Level 2	1873 (13.8)	134 (13.3)	607 (13.7)		245 (13.4)	887 (14.2)		
Level 3	7278 (53.8)	452 (44.8)	2246 (50.6)		1019 (55.6)	3561 (57.0)		
Level 4	2668 (19.7)	258 (25.5)	1021 (23.0)		289 (15.8)	1100 (17.6)		
Level 5	1337 (9.9)	134 (13.3)	508 (11.4)		172 (9.4)	523 (8.4)		
Main reason for ED visit (*n* = 13,637)								
Bleeding	621 (4.6)	54 (5.3)	108 (2.4)	<0.01	310 (16.9)	149 (2.4)	<0.01	<0.01
Cardiovascular	1584 (11.6)	70 (6.9)	665 (14.8)	<0.01	104 (5.7)	745 (11.8)	<0.01	0.19
Fall	1020 (7.5)	11 (1.1)	58 (1.3)	0.59	238 (12.9)	713 (11.3)	0.06	<0.01
Hepatic/gastrointestinal	2756 (20.2)	210 (20.7)	1499 (33.3)	<0.01	185 (10.1)	862 (13.7)	<0.01	<0.01
Malaise and fatigue	2417 (17.7)	143 (14.1)	612 (13.6)	0.69	353 (19.2)	1309 (20.8)	0.13	<0.01
Neurologic	942 (6.9)	167 (16.5)	278 (6.2)	<0.01	156 (8.5)	341 (5.4)	<0.01	<0.01
Respiratory	1776 (13.0)	58 (5.7)	404 (9.0)	<0.01	177 (9.6)	1137 (18.1)	<0.01	<0.01
Rheumatologic	514 (3.8)	30 (3.0)	252 (5.6)	<0.01	22 (1.2)	210 (3.3)	<0.01	<0.01
Trauma	475 (3.5)	21 (2.1)	143 (3.2)	0.06	56 (3.0)	255 (4.1)	0.05	0.12
Others	1532 (11.2)	251 (24.7)	493 (11.0)		238 (12.9)	566 (9.0)	<0.01	<0.01
Outcome data								
Disposition (*n* = 13,600)				<0.01			<0.01	<0.01
Discharge	7842 (57.7)	680 (67.1)	3241 (72.4)		785 (42.7)	3136 (50.0)		
Hospitalization	5692 (41.9)	333 (32.8)	1232 (27.5)		1021 (55.6)	3106 (49.5)		
Death	66 (0.5)	1 (0.1)	1 (0.0)		31 (1.7)	33 (0.5)		
Clinical-biological data								
Obesity (BMI > 30 kg/m^2^) (*n* = 11,342)	1577 (13.9)	103 (13.8)	601 (14.7)	0.54	167 (12.6)	707 (13.6)	0.34	0.43
Treated comorbidities (*n* = 13,653)								
Diabetes	2554 (18.7)	175 (17.2)	455 (10.1)	<0.01	457 (24.9)	1467 (23.3)	0.17	<0.01
Cardiovascular disorder	8386 (61.4)	342 (33.7)	1371 (30.5)	0.05	1588 (86.4)	5085 (80.8)	<0.01	<0.01
Active cancer	287 (2.1)	49 (4.8)	78 (1.7)	<0.01	57 (3.1)	103 (1.6)	<0.01	0.02
Mental or behavioural disorder	5995 (43.9)	404 (39.8)	1398 (31.1)	<0.01	978 (53.2)	3215 (51.1)	0.11	<0.01
Chronic respiratory disease	1806 (13.2)	77 (7.6)	506 (11.2)	<0.01	238 (12.9)	985 (15.6)	<0.01	<0.01
Kidney and hepatic function								
GFR (ml/min/1.73 m^2^) (*n* = 11,159)	72.9 (±30.5)	92.2 (±44.2)	93.6 (±23.7)	0.79	57.9 (±26.4)	62.0 (±24.6)	<0.01	<0.01
Kidney failure (GFR < 60) (*n* = 11,825)	3768 (31.9)	101 (13.0)	287 (7.9)	<0.01	859 (51.1)	2521 (44.0)	<0.01	<0.01
Increased AST and/or ALT (*n* = 6758)	1801 (26.6)	153 (31.1)	614 (27.0)	0.06	200 (22.1)	834 (27.0)	<0.01	<0.01
Electrolyte disorders								
Dysnatraemia (*n* = 12,172)	2330 (19.1)	148 (18.6)	478 (12.5)	<0.01	462 (27.0)	1242 (21.2)	<0.01	<0.01
Dyskalaemia (*n* = 11,603)	2981 (25.7)	180 (23.4)	628 (17.2)	<0.01	590 (36.2)	1583 (28.5)	<0.01	<0.01
Blood cell count disturbances								
Anaemia (*n* = 12,171)	4115 (33.8)	228 (28.8)	720 (18.8)	<0.01	903 (52.4)	2264 (38.8)	<0.01	<0.01
Leucocytosis (*n* = 12,104)	4101 (33.9)	270 (34.2)	1200 (31.6)	0.15	615 (35.9)	2016 (34.8)	0.38	0.4
Thrombocytopenia (*n* = 12,117)	1200 (9.9)	89 (11.3)	303 (8.0)	<0.01	204 (11.9)	604 (10.4)	0.08	0.66

Data are the mean (±SD), or *n* (%). French Emergency Nurses Classification in Hospital scale (FRENCH): Level 1: Immediately life-threatening; Level 2: Marked impairment of a vital organ, or imminently life-threatening, or functionally disabling traumatic lesion; Level 3: Functional impairment, or organic lesions likely to deteriorate within 24 h, or complex medical situation justifying the use of several hospital resources; Level 4: Stable, non-complex functional impairment or organic lesions, but justifying the urgent use of at least one hospital resource; Level 5: No functional impairment or organic lesion justifying the use of hospital resources. ADE, adverse drug event; BMI, body mass index; ALT, alanine aminotransferase; AST, aspartate aminotransferase; ED, emergency department; GFR, glomerular filtration rate, estimated by MDRD or CKD-EPI formula. Kidney failure: this variable represents the number of patients with a GFR estimate <60 mL/min/1.73 m^2^.

**Table 2:** Therapeutic data by age group and presence/absence of an ADE.

	Study Population (*n* = 13,653)	Group 1			Group 2			Comparison of ADE Population (Group 1 vs. Group 2) *p*-Value
		ADE Population (*n* = 1016)	Non-ADE Population (*n* = 4502)	*p*-Value	ADE Population (*n* = 1839)	Non-ADE Population (*n* = 6296)	*p*-Value	
Number of treatments (*n* = 13,653)	6.5 (±4.0)	5.1 (±3.8)	4.4 (±3.5)	<0.01	8.4 (±3.7)	7.7 (±3.8)	<0.01	<0.01
Treatment management								
Independent management of medications (*n* = 13,609)	9616 (70.7)	892 (87.9)	4158 (93.0)	<0.01	997 (54.2)	3569 (56.8)	0.05	<0.01
Self-medication (*n* = 13,653)	3808 (27.9)	248 (24.4)	2078 (46.2)	<0.01	242 (13.2)	1240 (19.7)	<0.01	<0.01
Compliance with treatment (*n* = 13,639)	8176 (59.9)	386 (38.1)	2430 (54.0)	<0.01	1088 (59.2)	4272 (67.9)	<0.01	<0.01
Treatment omission (*n* = 13,639)	1162 (8.5)	186 (18.3)	432 (9.6)	<0.01	132 (7.2)	412 (6.6)	0.35	<0.01
Self-modification of treatment duration (*n* = 13,639)	638 (4.7)	132 (13.0)	231 (5.1)	<0.01	93 (5.1)	182 (2.9)	<0.01	<0.01
Self-modification of treatment dose (*n* = 13,639)	760 (5.6)	136 (13.4)	274 (6.1)	<0.01	109 (5.9)	241 (3.8)	<0.01	<0.01
Specific drug classes (*n* = 13,653)								
A02. Drugs for acid-related disorders	5008 (36.7)	242 (23.8)	1104 (24.5)	0.64	813 (44.2)	2849 (45.3)	0.43	<0.01
A10. Drugs used in diabetes	2554 (18.7)	175 (17.2)	455 (10.1)	<0.01	457 (24.9)	1467 (23.3)	0.17	<0.01
B01. Antithrombotic agents	6110 (44.8)	208 (20.5)	794 (17.6)	0.03	1384 (75.3)	3724 (59.1)	<0.01	<0.01
C03. Diuretics	3056 (22.4)	105 (10.3)	263 (5.8)	<0.01	716 (38.9)	1972 (31.3)	<0.01	<0.01
C07. Β-blocking agents	3451 (25.3)	146 (14.4)	511 (11.4)	<0.01	731 (39.7)	2063 (32.8)	<0.01	<0.01
C09. Agents acting on the renin-angiotensin system	4640 (34.0)	179 (17.6)	705 (15.7)	0.12	902 (49.0)	2854 (45.3)	<0.01	<0.01
C10. Lipid-modifying agents	3464 (25.4)	136 (13.4)	610 (13.5)	0.89	629 (34.2)	2089 (33.2)	0.41	<0.01
H02. Corticosteroids for systemic use	982 (7.2)	92 (9.1)	314 (7.0)	0.02	142 (7.7)	434 (6.9)	0.22	0.21
J01. Antibacterial drugs for systemic use	1645 (12.0)	145 (14.3)	489 (10.9)	<0.01	252 (13.7)	759 (12.1)	0.06	0.67
L01. Antineoplastic agents	287 (2.1)	49 (4.8)	78 (1.7)	<0.01	57 (3.1)	103 (1.6)	<0.01	0.02
L04. Immunosuppressants	217 (1.6)	29 (2.9)	97 (2.2)	0.18	25 (1.4)	66 (1.0)	0.26	<0.01
M01. Anti-inflammatory and antirheumatic products	1493 (10.9)	146 (14.4)	729 (16.2)	0.15	139 (7.6)	479 (7.6)	0.94	<0.01
N02. Analgesics	6655 (48.7)	379 (37.3)	2352 (52.2)	<0.01	840 (45.7)	3084 (49.0)	<0.01	<0.01
N04. Anti-Parkinson drugs	583 (4.3)	34 (3.3)	77 (1.7)	<0.01	108 (5.8)	364 (5.8)	0.88	<0.01
N05. Psycholeptics	4746 (34.8)	363 (35.7)	1170 (26.0)	<0.01	767 (41.7)	2446 (38.9)	0.03	<0.01
N06. Psychoanaleptics	3372 (24.7)	185 (18.2)	718 (15.9)	0.08	572 (31.1)	1897 (30.1)	0.42	<0.01
Inappropriate medications (*n* = 13,653)								
According to Beers Criteria				<0.01			<0.01	<0.01
Always	3795 (27.8)	282 (27.8)	912 (20.3)		639 (34.7)	1962 (31.2)		
Conditionally	5479 (40.1)	384 (37.8)	1548 (34.4)		844 (45.9)	2703 (42.9)		
According to Laroche’s list				<0.01			0.03	<0.01
Always	3303 (24.2)	298 (29.3)	1016 (22.6)		454 (24.7)	1535 (24.4)		
Conditionally	2203 (16.1)	89 (8.8)	346 (7.7)		437 (23.8)	1331 (21.1)		
According to PIM-EU7 list				<0.01			<0.01	<0.01
Always	6331 (46.4)	448 (44.1)	1573 (34.9)		1081 (58.8)	3229 (51.3)		
Conditionally	3058 (22.4)	166 (16.3)	917 (20.4)		409 (22.2)	1566 (24.9)		
According to at least one								
Always	7745 (56.7)	535 (52.7)	1935 (43.0)	<0.01	1305 (71.0)	3970 (63.1)	<0.01	<0.01
Always or conditionally	10,407 (76.2)	731 (71.9)	2857 (63.5)	<0.01	1616 (87.9)	5203 (82.6)	<0.01	<0.01
Anticholinergic agents (*n* = 13,653)								
According to ARS	2628 (19.2)	212 (20.9)	720 (16.0)	<0.01	391 (21.3)	1305 (20.7)	0.62	0.8
According to ADS	7570 (55.4)	590 (58.1)	1817 (40.4)	<0.01	1321 (71.8)	3842 (61.0)	<0.01	<0.01
According to ACB	6816 (49.9)	495 (48.7)	1546 (34.3)	<0.01	1239 (67.4)	3536 (56.2)	<0.01	<0.01

Data are the mean (±SD), or *n* (%). ACB, anticholinergic burden; ADEs, adverse drug events; ADS, anticholinergic drug scale; ARS, anticholinergic risk scale; PIMs, potentially inappropriate medications.

**Table 3:** Characteristics of ADEs by age group.

	Group 1 (*n* = 1016)	Group 2 (*n* = 1839)	*p*-Value
ADE Symptoms (*n* = 2855)			
Bleeding	93 (9.2)	648 (35.2)	<0.01
Cardiovascular disorders	78 (7.7)	189 (10.3)	0.02
Fall	6 (0.6)	56 (3.0)	<0.01
Gastrointestinal disorders	174 (17.1)	121 (6.6)	<0.01
Hematology and coagulation test abnormalities	38 (3.7)	165 (9.0)	<0.01
Infections	56 (5.5)	36 (2.0)	<0.01
Malaise and fatigue	44 (4.3)	56 (3.0)	0.07
Metabolic disorders	129 (12.7)	242 (13.2)	0.72
Neurologic disorders	203 (20.0)	181 (9.8)	<0.01
Psychiatric disorders	31 (3.1)	14 (0.8)	<0.01
Respiratory disorders	24 (2.4)	35 (1.9)	0.41
Skin disorders	77 (7.6)	31 (1.7)	<0.01
Urologic or renal disorders	24 (2.4)	40 (2.2)	0.75
Others	39 (3.8)	25 (1.4)	<0.01
ADE Categories (*n* = 2847)			
Direct ADR	478 (47.2)	801 (43.7)	0.07
Participating ADR	193 (19.1)	816 (44.5)	<0.01
Noncompliance with drug prescription	342 (33.8)	217 (11.8)	<0.01
ADE Severity (*n* = 2855)			
Spontaneous regression	75 (7.4)	110 (6.0)	0.15
Regression after symptomatic treatment	590 (58.1)	722 (39.3)	<0.01
Hospitalization with no life threat	261 (25.7)	816 (44.4)	<0.01
Hospitalization with life-threatening risk	47 (4.6)	111 (6.0)	0.11
Death	1 (0.1)	31 (1.7)	<0.01
Undetermined	42 (4.1)	49 (2.7)	0.03

Data are presented as *n* (%). ADE, adverse drug event; ADR, adverse drug reaction.

**Table 4:** Multivariable analyses of variables associated with ADEs.

	Group 1			Group 2		
Variables	Odds Ratio	CI 95%	*p*-Value	Odds Ratio	CI 95%	*p*-Value
Admission data						
ED unit of inclusion						
Emergency critical care or short-stay hospitalization unit (vs. Observation emergency unit)	2.31	1.80–2.96	<0.01	1.78	1.52–2.08	<0.01
FRENCH Triage Scale (vs. other levels)						
Level 1				1.53	1.13–2.06	<0.01
Main reason for ED visit (vs. other reasons)						
Respiratory				0.43	0.35–0.52	<0.01
Hepatic/gastrointestinal				0.79	0.65–0.96	0.02
Cardiovascular				0.4	0.33–0.52	<0.01
Neurologic	2.7	2.10–3.47	<0.01	1.48	1.18–1.86	<0.01
Bleeding	2.48	1.71–3.60	<0.01	6.43	5.12–8.07	<0.01
Rheumatologic				0.27	0.15–0.49	<0.01
Clinical-biological data						
Kidney failure (GFR <60 vs. ≥60 mL/min/1.73 m^2^)	1.44	1.11–1.88	<0.01			
Dysnatraemia (vs. normal)				1.27	1.10–2.49	<0.01
Dyskalaemia (vs. normal)				1.2	1.15–1.48	<0.01
Therapeutic data						
Presence of specific drug classes (vs. other medication types)						
A10. Drugs used in diabetes	1.76	1.40–2.20	<0.01			
B01. Antithrombotic agents				1.89	1.64–2.17	<0.01
C07. Β-blocking agents				1.2	1.06–1.37	<0.01
L01. Antineoplastic agents	4.34	2.88–6.56	<0.01	1.97	1.34–2.91	<0.01
N02. Analgesics	0.55	0.46–0.65	<0.01	0.83	0.74–0.94	<0.01
Presence of always inappropriate medications according to at least one list (yes vs. under condition and no)				1.27	1.10–1.45	<0.01
Presence of anticholinergic medications according to ADS (vs. absence)	1.92	1.63–2.29	<0.01	1.46	1.27–1.68	<0.01
Compliance with treatment (yes vs. no)	0.55	0.46–0.65	<0.01	0.74	0.65–0.84	<0.01
Self-modification of treatment duration (yes vs. no)	1.98	1.51–2.61	<0.01	1.73	1.29–2.31	<0.01

CI, confidence intervals; ED, emergency department; GFR, glomerular filtration rate; French Emergency Nurses Classification in Hospital scale (FRENCH), level 1: Immediately life-threatening.

## Data Availability

The data analyzed during the current study are not publicly available due to ethical restrictions but are available from the corresponding author upon reasonable request.

## Supplementary Materials

The following supporting information can be downloaded at: https://www.mdpi.com/article/10.3390/jcm11195731/s1, Supplementary Figure S1: ADE Detection process, organization and analysis summary; Supplementary Table S1: Variables tested for inclusion in the multivariate models; Supplementary Table S2: General and therapeutic data in the total study population, Group 1 and Group 2, Supplementary Table S3: Distribution of medication involvement in ADEs and medication ADE rate by age group. Supplementary Table S4: Distribution of medications.
