# Supplementary material for: Frequency, Characteristics, and Predictive Factors of Adverse Drug Events in an Adult Emergency Department according to Age: A Cross-Sectional Study
Source: J Clin Med. 2022 Sep 27;11(19):5731. doi: 10.3390/jcm11195731 (PMC9572040; doi:10.3390/jcm11195731)

Supplementary Figure S1: ADE Detection process, organization and analysis summary. ADE, Adverse drug event. ED, Emergency department.

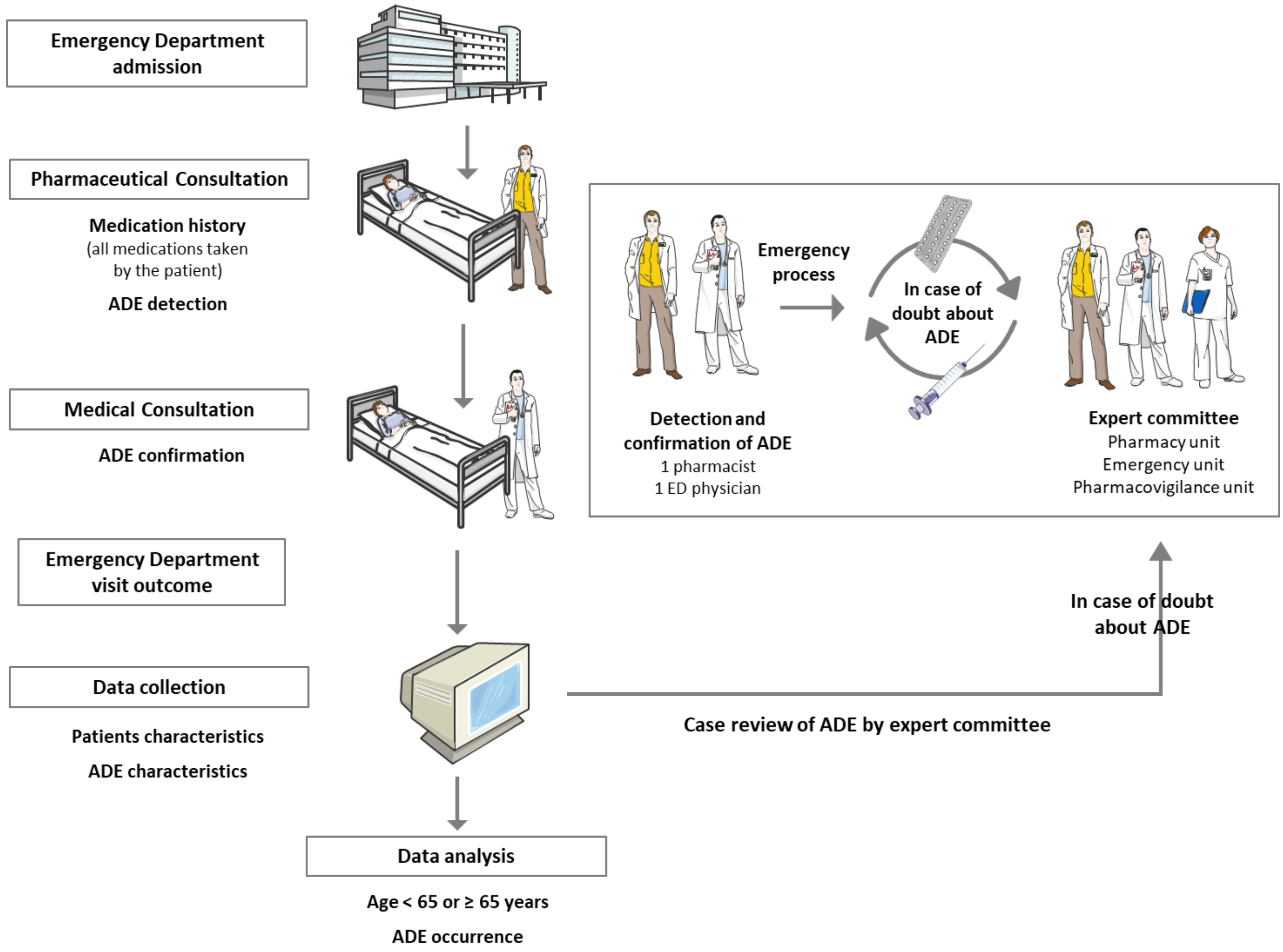

Supplement: Supplementary file 1 [file jcm-11-05731-s001.zip › Supplementary Figure S1.pdf]
