# Supplementary material for: Frequency, Characteristics, and Predictive Factors of Adverse Drug Events in an Adult Emergency Department according to Age: A Cross-Sectional Study
Source: J Clin Med. 2022 Sep 27;11(19):5731. doi: 10.3390/jcm11195731 (PMC9572040; doi:10.3390/jcm11195731)
Supplement: Supplementary file 1 [file jcm-11-05731-s001.zip › Supplementary Table S1.pdf]

Supplementary Table S1: Variables tested for inclusion in the multivariate models

	Group 1	Group 2
**Sociodemographic data**		
Age older than 75 years *(versus younger than 75 years)*		×
Male gender *(versus female)*	×	×
Living in an institution *(versus home)*	×	
**Admission data**		
ED unit of inclusion		
Emergency critical care or Short-stay hospitalization unit *(vs. Observation emergency unit)*	×	×
FRENCH Triage Scale *(vs. other levels)*		
Level 1	×	×
Main reason for ED visit *(versus other reasons)*		
Bleeding	×	×
Cardiovascular	×	×
Fall		×
Hepatic gastrointestinal	×	×
Neurologic	×	×
Respiratory	×	×
Rheumatologic	×	×
Trauma	×	×
**Clinical-biological data**		
Elevated creatinine serum level *(>84 µmol/l in women or 104 µmol/l in men versus lower values)*	×	×
Kidney failure *(GFR<60 versus ≥60 ml/min/1.73m²)*	×	×
Dysnatremia *(versus normal)*	×	×
Dyskaliemia *(versus normal)*	×	×
Anemia *(versus normal)*	×	×
Thrombocytopenia *(versus normal)*	×	×
**Therapeutic data**		
Presence of specific drug classes *(vs other medication types)*		
A10. Drugs used in diabetes	×	
B01. Antithrombotic agents	×	×
C03. Diuretics	×	×
C07. Β-blocking agents	×	×
C09. Agents acting on the renin-angiotensin system		×
H02. Corticosteroids for systemic use	×	
J01. Antibacterial drugs for systemic use	×	
L01. Antineoplastic agents	×	×
N02. Analgesics	×	×
N04. Anti-Parkinson drugs	×	
N05. Psycholeptics	×	×
Presence of inappropriate medications		
According to Beers criteria (*yes vs conditionally or no)*	×	×
According to Beers criteria (*yes or conditionally versus no)*	×	×
According to Laroche (*yes vs conditionally or no)*	×	×
According to Laroche (*yes or conditionally versus no)*	×	
According to PIM-EU7 (*yes vs conditionally or no)*	×	×
According to PIM-EU7 (*yes or conditionally versus no)*	×	×
According to at least one list *(yes vs under condition and no)*	×	×
According to at least one list (*yes or conditionally versus no)*	×	×
Presence of anticholinergic medications *(vs absence)*		
According to ARS	×	
According to ACB	×	×
According to ADS	×	×
Treatment management		
Self-medication *(yes vs no)*	×	×
Compliance with treatment *(yes vs no)*	×	×
Treatment omission *(yes vs no)*	×	
Self-modification of treatment duration *(yes vs no)*	×	×
Self-modification of treatment dose *(yes vs no)*	×	×
Number of treatments	×	×

ACB, anticholinergic burden; ADE, adverse drug event; ADS, Anticholinergic Drug Scale; ARS, Anticholinergic Risk Scale; FRench Emergency Nurses Classification in Hospital scale (FRENCH), level 1: Immediately life-threatening; GFR, glomerular filtration rate; PIM potentially inappropriate medications.
