# Supplementary material for: Frequency, Characteristics, and Predictive Factors of Adverse Drug Events in an Adult Emergency Department according to Age: A Cross-Sectional Study
Source: J Clin Med. 2022 Sep 27;11(19):5731. doi: 10.3390/jcm11195731 (PMC9572040; doi:10.3390/jcm11195731)
Supplement: Supplementary file 1 [file jcm-11-05731-s001.zip › Supplementary Table S2.pdf]

Supplementary Table S2: General and therapeutic data in the total study population, Group 1 and Group 2

Characteristics	Study population (n=13,653)	Group 1 (n=5,518)	Group 2 (n=8,135)	*p-value*
**Sociodemographic data**				
Age (n=13,653)	66.1 (± 14.1)	43.8 (± 14.1)	81.2 (± 14.9)	*<0.01*
Gender (n=13,653) - Female	7,135 (52.26)	2,789 (50.5)	4,346 (53.4)	*0.01*
Lifestyle (n=13,620)				*<0.01*
At home	12,120 (89.0)	5,323 (96.9)	6,797 (83.6)	
In institution	1,500 (11.0)	168 (3.1)	1,332 (16.4)	
**Admission data**				
ED unit of inclusion (n=13,635)				*<0.01*
Emergency critical care unit	455 (3.34)	118 (2.1)	337 (4.2)	
Observation emergency unit	11,983 (87.88)	5,094 (92.5)	6,889 (84.7)	
Short-stay hospitalization unit	1,197 (8.78)	293 (5.3)	904 (11.1)	
FRENCH Triage Scale (n=13,528)				*<0.01*
Level 1	372 (2.75)	91 (1.7)	281 (3.5)	
Level 2	1,873 (13.85)	741 (13.6)	1,132 (14.0)	
Level 3	7,278 (53.80)	2,698 (49.5)	4,580 (56.7)	
Level 4	2,668 (19.72)	1,279 (23.5)	1,389 (17.2)	
Level 5	1,337 (9.88)	642 (11.8)	695 (8.6)	
Main reason for ED visit (n=13,637)				
Bleeding	621 (4.6)	162 (2.9)	459 (5.7)	*<0.01*
Cardiovascular	1,584 (11.6)	735 (13.3)	849 (10.5)	*<0.01*
Fall	1,020 (7.5)	69 (1.3)	951 (11.7)	*<0.01*
Hepatic/gastrointestinal	2,756 (20.2)	1,709 (31.0)	1047 (12.9)	*<0.01*
Malaise and fatigue	2,417 (17.7)	755 (13.7)	1,662 (20.5)	*<0.01*
Neurologic	942 (6.9)	445 (8.1)	497 (6.1)	*<0.01*
Respiratory	1,776 (13.0)	462 (8.4)	1314 (16.2)	*<0.01*
Rheumatologic	514 (3.8)	282 (5.1)	232 (2.9)	*<0.01*
Trauma	475 (3.5)	164 (3.0)	311 (3.8)	*0.01*
Others	1,532 (11.2)	728 (13.2)	804 (9.9)	*<0.01*
**Outcome data**				
Disposition (n=13,600)				*<0.01*
Discharge	7842 (57.7)	3,921 (71.5)	3,921 (48.3)	
Hospitalization	5692 (41.9)	1,565 (28.5)	4,127 (50.9)	
Death	66 (0.5)	2 (0.0)	64 (0.8)	
**Clinical-biological data**				
Obesity (BMI>30 kg/m²) (n=11,342)	1,577 (13.9)	704 (14.6)	874 (13.4)	*0.08*
Treated comorbidities (n=13,653)				
Diabetes	2,554 (18.7)	630 (11.4)	1,924 (23.7)	*<0.01*
Cardiovascular disorder	8,386 (61.4)	1,713 (31.0)	6,673 (82.0)	*<0.01*
Active cancer	287 (2.1)	127 (2.3)	160 (2.0)	*0.18*
Mental or behavioural disorder	5,995 (43.9)	1,802 (32.7)	4,193 (51.5)	*<0.01*
Chronic respiratory disease	1,806 (13.2)	583 (10.6)	1,223 (15.0)	*<0.01*
Kidney and hepatic function				
GFR (ml/min/1.73m²) (n=11,159)	72.9 (± 30.5)	93.4 (± 28.2)	61.1 (± 25.0)	*<0.01*
Kidney failure (GFR<60) (n=11,825)	3,768 (31.9)	388 (8.8)	3,380 (45.6)	*<0.01*
Increased AST and/or ALT (n=6,758)	1,801 (26.6)	767 (27.7)	1,034 (25.9)	*0.11*
Electrolyte disorders				
Dysnatraemia (n=12,172)	2,330 (19.1)	626 (13.6)	1,704 (22.5)	*<0.01*
Dyskalaemia (n=11,603)	2,981 (25.7)	808 (18.3)	2,173 (30.3)	*<0.01*
Blood cell count disturbances				
Anaemia (n=12,171)	4,115 (33.8)	948 (20.6)	3,167 (41.9)	*<0.01*
Leucocytosis (n=12,104)	4,101 (33.9)	1,470 (32.0)	2,631 (35.0)	*<0.01*
Thrombocytopenia (n=12,117)	1,200 (9.9)	392 (8.5)	808 (10.7)	*<0.01*
**Therapeutic data**				
Number of treatments (n=13,653)	6.5 ± 4.0	4.6 ± 3.5	7.9 ± 3.8	*<0.01*
Treatment management				
Independent management of medications (n=13,609)	9,616 (70.7)	5,050 (92.1)	4,566 (56.2)	*<0.01*
Self-medication (n=13,653)	3,808 (27.9)	2,326 (42.2)	1,484 (18.2)	*<0.01*

Characteristics	Study population (n=13,653)	Group 1 (n=5,518)	Group 2 (n=8,135)	*p-value*
Compliance with treatment (n=13,639)	8,176 (59.9)	2,816 (51.1)	5,360 (66.0)	*<0.01*
Treatment omission (n=13,639)	1,162 (8.5)	618 (11.2)	544 (6.7)	*<0.01*
Self-modification of treatment duration (n=13,639)	638 (4.7)	363 (6.6)	275 (3.4)	*<0.01*
Self-modification of treatment dose (n=13,639)	760 (5.6)	410 (7.4)	350 (4.3)	*<0.01*
Specific drug classes (n=13,653)				
A02. Drugs for acid-related disorders	5,008 (36.7)	1,346 (54.4)	3,662 (45.0)	*<0.01*
A10. Drugs used in diabetes	2,554 (18.7)	630 (11.4)	1,924 (23.7)	*<0.01*
B01. Antithrombotic agents	6,110 (44.8)	1,002 (18.2)	5,108 (62.8)	*<0.01*
C03. Diuretics	3,056 (22.4)	368 (6.7)	2,688 (33.0)	*<0.01*
C07. B-blocking agents	3,451 (25.3)	657 (11.9)	2,794 (34.3)	*<0.01*
C09. Agents acting on the renin-angiotensin system	4,640 (34.0)	884 (16.0)	3,756 (46.2)	*<0.01*
C10. Lipid-modifying agents	3,464 (25.4)	746 (13.5)	2,718 (33.4)	*<0.01*
H02. Corticosteroids for systemic use	982 (7.2)	406 (7.4)	576 (7.1)	*0.54*
J01. Antibacterial drugs for systemic use	1,645 (12.0)	634 (11.5)	1,011 (12.4)	*0.10*
L01. Antineoplastic agents	287 (2.1)	127 (2.3)	160 (2.0)	*0.18*
L04. Immunosuppressants	217 (1.6)	126 (2.3)	91 (1.1)	*<0.01*
M01. Anti-inflammatory and antirheumatic products	1,493 (10.9)	875 (15.9)	618 (7.6)	*<0.01*
N02. Analgesics	6,655 (48.7)	2,731 (49.5)	3,924 (48.2)	*0.15*
N04. Anti-Parkinson drugs	583 (4.3)	111 (2.0)	472 (5.8)	*<0.01*
N05. Psycholeptics	4,746 (34.8)	1,533 (27.8)	3,213 (39.5)	*<0.01*
N06. Psychoanaleptics	3,372 (24.7)	903 (16.4)	2,469 (30.4)	*<0.01*
Inappropriate medications (n=13,653)				
According to Beers Criteria				*<0.001*
Always	3,795 (27.8)	1,194 (21.6)	2,601 (32.0)	
Conditionally	5,479 (40.1)	1,932 (35.0)	3,547 (43.6)	
According to Laroche's list				*<0.001*
Always	3,303 (24.2)	1,314 (23.8)	1,989 (24.5)	
Conditionally	2,203 (16.1)	435 (7.9)	1,768 (21.7)	
According to PIM-EU7 list				*<0.001*
Always	6,331 (46.4)	2,021 (36.6)	4,310 (53.0)	
Conditionally	3,058 (22.4)	1,083 (19.6)	1,975 (24.3)	
According to at least one				
Always	7,745 (56.7)	2,470 (44.8)	5,275 (64.8)	*<0.001*
Always or conditionally	10,407 (76.2)	3,588 (65.0)	6,819 (83.8)	*<0.001*
Anticholinergic agents (n=13,653)				
According to ARS	2,628 (19.2)	932 (16.9)	1,696 (20.8)	*<0.001*
According to ADS	7,570 (55.4)	2,407 (43.6)	5,163 (63.5)	*<0.001*
According to ACB	6,816 (49.9)	2,041 (37.0)	4,775 (58.7)	*<0.001*

Data are the mean (± SD), or n (%)

FRench Emergency Nurses Classification in Hospital scale (FRENCH): Level 1: Immediately life-threatening; Level 2: Marked impairment of a vital organ, or imminently life-threatening, or functionally disabling traumatic lesion; Level 3: Functional impairment, or organic lesions likely to deteriorate within 24h, or complex medical situation justifying the use of several hospital resources; Level 4: Stable, non-complex functional impairment or organic lesions, but justifying the urgent use of at least one hospital resource; Level 5: No functional impairment or organic lesion justifying the use of hospital resources.

ACB, anticholinergic burden; ADS, Anticholinergic Drug Scale; ARS, Anticholinergic Risk Scale; BMI, body mass index; ALT, alanine aminotransferase; AST, aspartate aminotransferase; ED, emergency department; GFR, glomerular filtration rate, estimated by MDRD or CKD-EPI formula; PIMs, potentially inappropriate medications.

Kidney failure: this variable represents the number of patients with a GFR estimate <60 ml/min/1.73m².
