# Supplementary material for: Frequency, Characteristics, and Predictive Factors of Adverse Drug Events in an Adult Emergency Department according to Age: A Cross-Sectional Study
Source: J Clin Med. 2022 Sep 27;11(19):5731. doi: 10.3390/jcm11195731 (PMC9572040; doi:10.3390/jcm11195731)
Supplement: Supplementary file 1 [file jcm-11-05731-s001.zip › Supplementary Table S3.pdf]

Supplementary Table S2: Distribution of medication involvement in ADEs and medication ADE rate by age group.

	Group 1		Group 2	
	**Involved in an ADE** (n=1,398)	**ADE rate** (%)	**Involved in an ADE** (n=2,449)	**ADE rate** (%)
**A. Alimentary tract and metabolism**	**197 (14.1)**	**3.8**	**190 (7.8)**	**1.4**
A10. Drugs used in diabetes	156 (11.2)	13.1	144 (5.9)	4.3
Others	41 (2.9)	1.0	46 (1.9)	0.5
**B. Blood and blood forming organs**	**124 (8.9)**	**8.2**	**899 (36.7)**	**13.0**
B01. Antithrombotic agents	122 (8.7)	10.1	894 (36.5)	15.2
Others	2 (0.1)	0.6	5 (0.2)	0.5
**C. Cardiovascular system**	**133 (9.5)**	**3.8**	**455 (18.6)**	**2.7**
C01. Cardiac therapy	7 (0.5)	2.7	43 (1.8)	2.0
C03. Diuretics	40 (2.9)	9.0	148 (6.0)	5.0
C07. Β-blocking agents	24 (1.7)	3.6	67 (2.7)	2.4
C08. Calcium channel blockers	14 (1.0)	4.3	26 (1.1)	1.4
C09. Agents acting on the renin-angiotensin system	38 (2.7)	4.2	147 (6.0)	3.8
Others	10 (0.7)	1.1	24 (1.0)	0.7
**G. Genitourinary system and sex hormones**	**15 (1.1)**	**2.3**	**29 (1.2)**	**1.6**
**H. Systemic hormonal preparations**	**44 (3.1)**	**5.2**	**41 (1.7)**	**2.3**
H02. Corticosteroids for systematic use	33 (2.4)	7.9	34 (1.4)	5.7
Others	11 (0.8)	2.6	7 (0.3)	0.6
**J. Anti-infective drugs for systemic use**	**108 (7.7)**	**11.1**	**99 (4.0)**	**7.6**
J01. Antibacterial drugs for systemic use	92 (6.6)	12.4	93 (3.8)	7.9
Others	16 (1.1)	7.0	6 (0.2)	4.5
**L. Antineoplastic and immunomodulating agents**	**76 (5.4)**	**18.3**	**74 (3.0)**	**15.0**
L01. Antineoplastic agents	54 (3.9)	30.5	58 (2.4)	29.7
L04. Immunosuppressants	17 (1.2)	9.2	12 (0.5)	11.0
Others	5 (0.4)	9.1	4 (0.2)	2.1
**M. Muscular-skeletal system**	**99 (7.1)**	**7.6**	**48 (2.0)**	**2.9**
M01. Anti-inflammatory and antirheumatic products	79 (5.7)	8.2	27 (1.1)	4.0
Others	20 (1.4)	5.8	21 (0.9)	2.2
**N. Nervous system**	**543 (38.8)**	**6.5**	**564 (23.0)**	**3.9**
N02. Analgesics	134 (9.6)	3.9	154 (6.3)	3.1
N03. Antiepileptic drugs	138 (9.9)	16.6	37 (1.5)	3.6
N04. Anti-Parkinson drugs	12 (0.9)	9.5	32 (1.3)	4.9
N05. Psycholeptics	180 (12.9)	6.9	221 (9.0)	4.8
N06. Psychoanaleptics	43 (3.1)	4.3	110 (4.5)	3.7
Others	36 (2.6)	12.6	10 (0.4)	2.2
**R. Respiratory system**	**33 (2.4)**	**1.8**	**32 (1.3)**	**1.1**
R03. Antiasthmatics	21 (1.5)	2.1	21 (0.9)	1.0
Others	12 (0.9)	1.4	11 (0.4)	2.2
**Others**	**26 (1.9)**	**4.1**	**18 (0.7)**	**0.8**

Data are presented as n (%) for medication involvement in an ADE (adverse drug event), classified by ATC (Anatomical Therapeutic Chemical) levels 1 and 2 (only for a frequency ≥ 1.0% in at least one of the age groups).
Medication ADE rates are determined by dividing the number of a medication's involvement in an ADE by the total number of prescriptions for that medication. ADE rates are presented as percentages.
