# Supplementary material for: Frequency, Characteristics, and Predictive Factors of Adverse Drug Events in an Adult Emergency Department according to Age: A Cross-Sectional Study
Source: J Clin Med. 2022 Sep 27;11(19):5731. doi: 10.3390/jcm11195731 (PMC9572040; doi:10.3390/jcm11195731)
Supplement: Supplementary file 1 [file jcm-11-05731-s001.zip › Supplementary Table S4.pdf]

Supplementary Table S3: Distribution of medications

		Total (n=89,095)	Group 1 (n=25,130)	Group 2 (n=63,965)
**A.**	**Alimentary tract and metabolism**	**18,530 (20.8)**	**5,151 (20.5)**	**13,379 (20.9)**
	A02. Drugs for acid-related disorders	5,439 (6.1)	1,507 (6.0)	3,932 (6.1)
	A03. Drugs for functional gastrointestinal disorders	1,609 (1.8)	849 (3.4)	760 (1.2)
	A06. Laxatives	2,359 (2.6)	381 (1.5)	1,978 (3.1)
	A10. Drugs used in diabetes	4,567 (5.1)	1,194 (4.8)	3,373 (5.3)
	A11. Vitamins	1,423 (1.6)	296 (1.2)	1,127 (1.8)
	A12. Supplementation	1,626 (1.8)	266 (1.1)	1,360 (2.1)
	Others	1,507 (1.7)	658 (2.6)	849 (1.3)
**B.**	**Blood and blood forming organs**	**8,414 (9.4)**	**1,516 (6.0)**	**6,898 (10.8)**
	B01. Antithrombotic agents	7,101 (8.0)	1,207 (4.8)	5,894 (9.2)
	Others	1,313 (1.5)	309 (1.2)	1,004 (1.6)
**C.**	**Cardiovascular system**	**20,413 (22.9)**	**3,513 (14.0)**	**16,900 (26.4)**
	C01. Cardiac therapy	2,361 (2.6)	256 (1.0)	2,105 (3.3)
	C03. Diuretics	3,420 (3.8)	444 (1.8)	2,976 (4.7)
	C07. Β-blocking agents	3,478 (3.9)	663 (2.6)	2,815 (4.4)
	C08. Calcium channel blockers	2,153 (2.4)	323 (1.3)	1,830 (2.9)
	C09. Agents acting on the renin-angiotensin system	4,745 (5.3)	912 (3.6)	3,833 (6.0)
	C10. Lipid-modifying agents	3,582 (4.0)	789 (3.1)	2,793 (4.4)
	Others	674 (0.8)	126 (0.5)	548 (0.9)
**G.**	**Genitourinary system and sex hormones**	**2,476 (2.8)**	**640 (2.6)**	**1,836 (2.9)**
	G04. Urological drugs	1,897 (2.1)	208 (0.8)	1,689 (2.6)
	Others	579 (0.6)	432 (1.7)	147 (0.2)
**H.**	**Systemic hormonal preparations**	**2,657 (3.0)**	**847 (3.4)**	**1,810 (2.8)**
	H02. Corticosteroids for systemic use	1,012 (1.1)	420 (1.7)	592 (0.9)
	H03. Thyroid therapy	1,551 (1.7)	384 (1.5)	1,167 (1.8)
	Others	94 (0.1)	43 (0.2)	51 (0.1)
**J.**	**Anti-infective drugs for systemic use**	**2,282 (2.6)**	**971 (3.9)**	**1,311 (2.0)**
	J01. Antibacterial drugs for systemic use	1919 (2.2)	742 (3.0)	1177 (1.8)
	Others	363 (0.4)	229 (0.9)	134 (0.2)
**L.**	**Antineoplastic and immunomodulating agents**	**909 (1.0)**	**416 (1.7)**	**493 (0.8)**
**M.**	**Muscular-skeletal system**	**2,944 (3.3)**	**1301 (5.2)**	**1,643 (2.6)**
	M01. Anti-inflammatory and antirheumatic products	1640 (1.8)	959 (3.8)	681 (1.1)
	Others	1304 (1.5)	342 (1.4)	962 (1.5)
**N.**	**Nervous system**	**22,850 (25.6)**	**8,323 (33.1)**	**14,527 (22.7)**
	N02. Analgesics	8,354 (9.4)	3,446 (13.7)	4908 (7.7)
	N03. Antiepileptic drugs	1,846 (2.1)	830 (3.3)	1016 (1.6)
	N05. Psycholeptics	7,255 (8.1)	2,628 (10.5)	4627 (7.2)
	N06. Psychoanaleptics	3,993 (4.5)	1,008 (4.0)	2985 (4.7)
	Others	1,402 (1.6)	411 (1.6)	991 (1.5)
**R.**	**Respiratory system**	**4,758 (5.3)**	**1,812 (7.2)**	**2,946 (4.6)**
	R03. Antiasthmatics	3,004 (3.4)	984 (3.9)	2,020 (3.2)
	R06. Antihistamines for systemic use	969 (1.1)	484 (1.9)	485 (0.8)
	Other	785 (0.9)	344 (1.4)	441 (0.7)
**S.**	**Sensory organs**	**1,095 (1.2)**	**150 (0.6)**	**945 (1.5)**
**Others**		**1,767 (2.0)**	**490 (1.9)**	**1,277 (2.0)**

Data are presented as n (%), classified by ATC (Anatomical Therapeutic Chemical) levels 1 and 2 (only for a frequency ≥ 1.5% in at least one of the age groups).
